# Engineered exosomes from different sources for cancer-targeted therapy

**DOI:** 10.1038/s41392-023-01382-y

**Published:** 2023-03-15

**Authors:** Menghui Zhang, Shengyun Hu, Lin Liu, Pengyuan Dang, Yang Liu, Zhenqiang Sun, Bingbing Qiao, Chengzeng Wang

**Affiliations:** 1 Department of Hepatobiliary and Pancreatic Surgery, The First Affiliated Hospital of Zhengzhou University, Zhengzhou, Henan 450001 China; 2 Department of Colorectal Surgery, The First Affiliated Hospital of Zhengzhou University, Zhengzhou, Henan 450001 China; 3 Henan Institute of Interconnected Intelligent Health Management, The First Affiliated Hospital of Zhengzhou University, Zhengzhou, Henan 450001 China; 4 Department of Ultrasound, The First Affiliated Hospital of Zhengzhou University, Zhengzhou, Henan 450001 China; 5 Department of Radiotherapy, Affiliated Cancer Hospital of Zhengzhou University, Henan Cancer Hospital, Zhengzhou, Henan 450001 China

**Keywords:** Drug development, Nanobiotechnology

## Abstract

Exosome is a subgroup of extracellular vesicles, which has been serving as an efficient therapeutic tool for various diseases. Engineered exosomes are the sort of exosomes modified with surface decoration and internal therapeutic molecules. After appropriate modification, engineered exosomes are able to deliver antitumor drugs to tumor sites efficiently and precisely with fewer treatment-related adverse effects. However, there still exist many challenges for the clinical translation of engineered exosomes. For instance, what sources and modification strategies could endow exosomes with the most efficient antitumor activity is still poorly understood. Additionally, how to choose appropriately engineered exosomes in different antitumor therapies is another unresolved problem. In this review, we summarized the characteristics of engineered exosomes, especially the spatial and temporal properties. Additionally, we concluded the recent advances in engineered exosomes in the cancer fields, including the sources, isolation technologies, modification strategies, and labeling and imaging methods of engineered exosomes. Furthermore, the applications of engineered exosomes in different antitumor therapies were summarized, such as photodynamic therapy, gene therapy, and immunotherapy. Consequently, the above provides the cancer researchers in this community with the latest ideas on engineered exosome modification and new direction of new drug development, which is prospective to accelerate the clinical translation of engineered exosomes for cancer-targeted therapy.

## Introduction

**Fig. 1:**
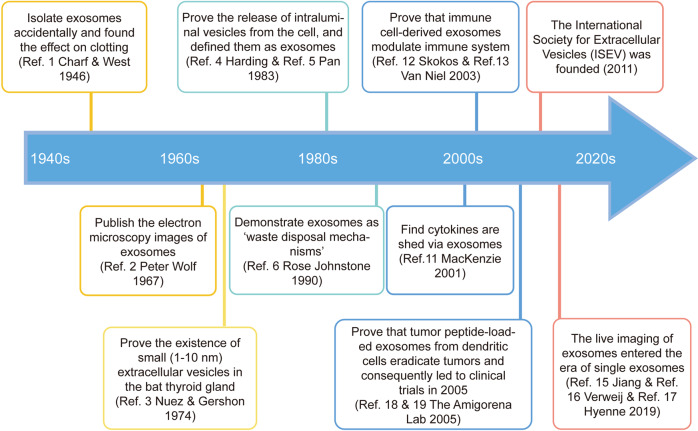
Timeline of exosomes milestones. In 1946, the finding of one clotting factor, similar to the thromboplastic protein, by Chargaff was considered as the beginning of the field of extracellular vesicles (EVs). The EV field emerged the exponential growth in the early 21st century. In 2011, the foundation of the International Society for Extracellular Vesicles (ISEV) promoted the development of exosomes extremely. In 2019, the imaging of exosomes entered the era of single exosomes in vivo

In 1946, Chargaff and West found a kind of clotting factor similar to the thromboplastic protein when they study anemia and hemophilia,^1^ which was considered the beginning of the field of extracellular vesicles (EV) biology. About 20 years later, Peter Wolf published electron microscopy images of these particles.^2^ In 1974, Nunez and Gershon proved the existence of small (1–10 nm) extracellular vesicles in the bat thyroid gland.^3^ In 1983, two studies proved the release of intraluminal vesicles from the cell, and defined them as exosomes, which were interpreted as the origins of the field.^4,5^ In addition to the discovery of exosomes, studies about the hallmarks of EVs also emerged. Rose Johnstone described exosomes as ‘waste disposal mechanisms’^6^ and that exosomes from reticulocytes were enzymatically active^7^ in 1990s. Meanwhile, several studies reported the quantification of exosomes, illustrating altered exosome numbers in plenty diseases.^8–10^ In the early 21st century, the increased interest in EVs led to the exponential growth of the field. For example, cytokines were found to be shed via exosomes in 2001.^11^ Two years later, immune cell-derived exosomes were proven to modulate the function of the immune system.^12,13^ In 2011, the International Society for Extracellular Vesicles (ISEV) was founded, which promoted the innovation and application of exosomes extremely. In 2014, the publication of ‘Minimal Information for Studies of EVs’ (MISEV) guidelines was an important event in the standardization of this field.^14^ In 2019, researchers detected single exosomes using new techniques,^15–17^ indicating the imaging of exosomes entered the era of single exosomes in vivo (Fig. 1).

Given the development and success of exosome research, engineered exosomes are attracting increasing attention, especially in cancer fields, due to their spatial and temporal targeting ability. In 2005, the Amigorena lab proved that exosomes from dendritic cells could eradicate tumors when loaded with tumor peptides^18^ and consequently led to clinical trials.^19^ Although natural exosomes can deliver antitumor drugs, they mostly retain in the liver or spleen rather than in tumor tissue. Additionally, the therapeutic efficacy is attenuated due to the heterogeneity and complicated components of natural EVs.^20^ Hence, engineering technologies confer exosomes more specifically targeting property and clinical translation potential. Nowadays, engineering strategies are various and different. It is generally divided into four categories: biological (targeting peptides), immunological (antibodies), physical (magnetic particles), and chemical modification (sodium bicarbonate). However, each engineering method has its own advantages and disadvantages and the ‘one-size-fits-all’ engineering approach does not exist yet. Hence, investigators chose to integrate multiple strategies to enhance the targeting ability of engineered exosomes. For instance, photo-activatable silencing extracellular vesicles (PASEVs) were loaded with siRNA against PAK4 (siPAK4) and photo-activatable reactive oxygen species (ROS)-sensitive polymer to form the nanocomplex core. Furthermore, the nanocomplex cores were camouflaged by extracellular vesicles from M1 macrophages. ROS-responsive release of siPAK4 not only silenced PAK4, but also modulated TME via immunogenic phototherapy21. Therefore, cancer immunotherapy was sensitized.

**Fig. 2:**
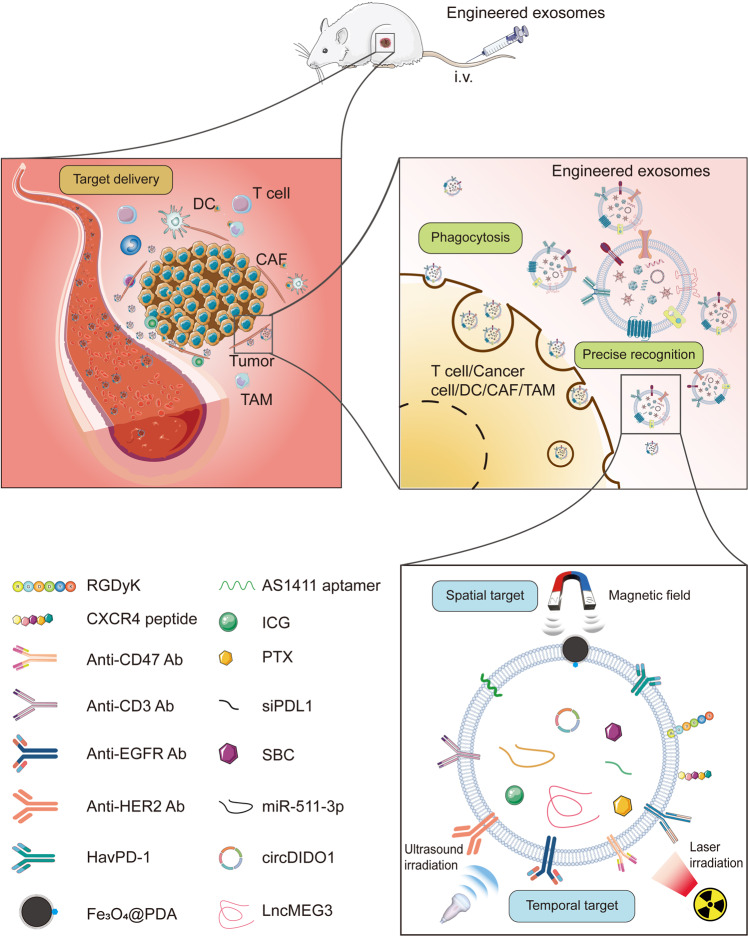
Schematic illustration of the engineered exosomes exhibiting antitumor effects on preclinical models. After intravenous injection, the engineered exosomes carrying the therapeutic molecules arrive at the tumor site under the guide of multiple targeting molecules or a magnetic field. Then the chemotherapeutic drugs, such as PTX, the ncRNAs, such as miR-551-3p, immune molecules, such siPDL1, were released into the tumor microenvironment (TME). Thereafter, the internalization of engineered exosomes led to tumor cell death, such as ferroptosis

In addition to strengthening the efficiency of antitumor therapies, engineered exosomes, as a new drug delivery platform, might alleviate the side effects during therapies, such as chemotherapy and radiotherapy. Injecting free chemotherapeutic intravenously is the mainstream of systemic therapy for cancers. However, due to the low targeting ability, this approach generally causes various therapy-related adverse effects (TRAEs) including leukocytopenia, hypothyroidism, and ALT or AST elevation. In order to address the above issues, engineered exosomes as a new drug delivery platform have attracted increasing attention. For example, melanoma-derived, doxorubicin (DOX)-loaded, and targeted peptide-decorated exosomes were developed to amplify the specific characteristics. The engineered exosomes preferentially target disease sites coupled with minimizing systemic off-target issues22. This review encapsulated the patterns of engineered exosomes and recent advances in the techniques of engineered exosomes. Additionally, the latest preclinical trials about the engineered exosome-based therapies were summarized, which were potential and beneficial for the translation of engineered exosomes in the future. (Fig. 2).

## The spatial-temporal patterns of engineered exosomes

### Engineered exosomes target tumor sites spatially

The exosomes decorated with specific targeted molecules, including peptides, can achieve more precise therapeutic cargo delivery. The miR-HER2 (human epidermal growth factor receptor 2)-loaded exosomes coated with ligands, which enables them to adhere to HER2 on the surface of human laryngeal carcinoma cells and preferentially enter HER2-positive cells. More importantly, the miR-HER2-loaded exosomes coated with additional peptide trigger more fierce tumoricidal effects than the exosomes without targeted peptide.^21^ Additionally, the plant-derived exosomes, especially medicinal herbs, also attracted the increasing attention of researchers due to the synergistic effects with low toxicity.^22^ For example, exosomes-like nanovesicles (ELNVs) derived from *Asparagus cochinchinensis* (Lour.) Merr. (ACNVs) were found to inhibit hepatocellular carcinoma (HCC) proliferation by activating apoptosis pathway.^23^ Despite the pronounced antitumor effects of natural ACNVs, the macropinocytosis of mononuclear phagocyte system (MPS), including in the liver and spleen, results in the quick clearance of exosomes in bloodstream and minimal accumulation within tumor area.^24^ Therefore, researchers decorated ACNVs with distearoyl phosphoethanolamine-polyethylene glycol 2000 (DSPE-PEG) to modify the pharmacokinetic profile of ACNVs. Consequently, in vivo, the PEG-ACNVs significantly suppressed tumor growth with fewer side effects and accumulated in the liver abundantly.^23^

In brief, the spatial targeting delivery of engineered exosomes depends on the targeted molecules on surface, which enables a heavier concentration of engineered exosomes inside tumors with lower in other healthy organs, such as liver, spleen, and kidney. However, there still lack ideal targeted molecules guiding exosomes to tumor sites and preventing exosomes from being absorbed by normal tissues as little as possible despite the mounting reports on the strategies of engineered exosomes.

### Engineered exosomes target tumor sites temporally

Decorating with specific targeted molecules enables engineered exosomes to deliver therapeutic cargos to tumor sites precisely. However, once engineered exosomes arrived at tumor tissue, the techniques fostering exosomes to exert antitumor effects are still insufficient. In order to address this challenge, exosomes were fused with drug-loaded thermosensitive liposomes,^25^ called hybrid therapeutic nanovesicles(hGLV). In vitro, hGLV demonstrated higher drug release with laser irradiation compared to the control without laser irradiation at 1 h and 6 h. Therefore, the hybrid-engineered exosomes enabled researchers to release antitumor drugs in a controllable temporal manner. This method releasing drugs controllably might inspire clinicians to manage the pharmacokinetics in the patients’ bodies. Similarly, the combination of chemotherapeutic and thermosensitive drugs also leads to pronounced antitumor effects in a controllable temporal manner. For example, curcumin and indocyanine green (ICG) co-loaded engineered exosomes effectively accumulated at glioma in vivo.^26^ Additionally, the permeability of the engineered exosomes membrane was significantly impacted by local hyperthermia, triggering drug release, and maintaining a sustained chemotherapeutic efficacy. Given triggering engineered exosomes to release therapeutic molecules controllably, compared to laser, ultrasound (US) possesses many advantages, including nonradiation and lower cost.^27^ The bone morphogenetic protein 7 (Bmp7) mRNA and chlorin e6 (Ce6)-loaded exosomes decorated with CP05-TK-mPEG on surface could be degraded locally by US and deliver Bmp7 mRNA to omental adipose tissue (OAT) of obese C57BL/6 mice precisely.^28^ Although the above approach was not conducted within tumor model, this strategy is innovative, which requires more experiments to confirm whether it is also suitable for cancers. Therefore, through additional physical impacts, such as laser and US irradiation, the temporal points when engineered exosomes unleash cargos can be controlled.

Taken together, engineered exosomes were demonstrated to deliver cargos to cancer sites spatially and release the bioactive therapeutic molecules temporally via targeted molecules on surface and additional physical irradiation. Over the last decades, molecular targeted drugs have extended the overall survival of patients with malignant diseases despite the poor spatially and temporally targeted properties. Engineered exosomes, serving as next-generation drug delivery platform,^29^ are expected to open new frontiers for modern drug delivery.

## The engineering strategies of exosomes that were used as a drug delivery platform

### The isolation of exosomes

The isolation of exosomes is significant to investigate their mechanisms of biological activity and application in cancer therapy. Conventional isolation methods of exosomes include ultracentrifugation, size-based filtration, size-exclusion chromatography, and polymer precipitation.^30^ Immunoaffinity methods exploit the interactions between proteins (antigens) and their antibodies, such as CD9, CD81, CD63 or cancer-specific proteins, which outweighs many other approaches in purity and yield.^31^ To improve this method, researchers used submicron-sized magnetic particles for immunoaffinity capture-magneto-immunocapture, which yields 10 to 15 times higher quantities of exosomes than ultracentrifugation.^32^ Additionally, these immunoaffinity methods have no volume limitations, endowing them with potential for large-scale clinical application. Although these approaches are useful in the lab, they generally suffer from various limitations in the clinical-grade application, such as burdensome procedures, high cost, and limited yield.

Recently, one new approach, microfluidic-based isolation technique, attracted increasing attentions for the microscale isolation, detection, and analysis of exosomes. This technique not only utilizes the usual separation determinants, like size, density, and immunoaffinity, but also exploits innovative sorting mechanisms, such as acoustic, electrophoretic, electromagnetic manipulations, nanowire-based traps (NTs), nano-sized deterministic lateral displacement (nano-DLD) and viscoelastic flow.^33,34^ For example, researchers fabricated and operated a micro-nanofluidic device that used cross-flow filtration to isolate and capture liposarcoma-derived exosomes. The device integrated the unit operations of size-based separation with CD63 antibody immunoaffinity-based capture to evaluate exosome-cargo content for liposarcoma. The method led to a five-fold increase in amount of critical liposarcoma-relevant extracellular vesicle cargo in 30 min.^35^

**Fig. 3:**
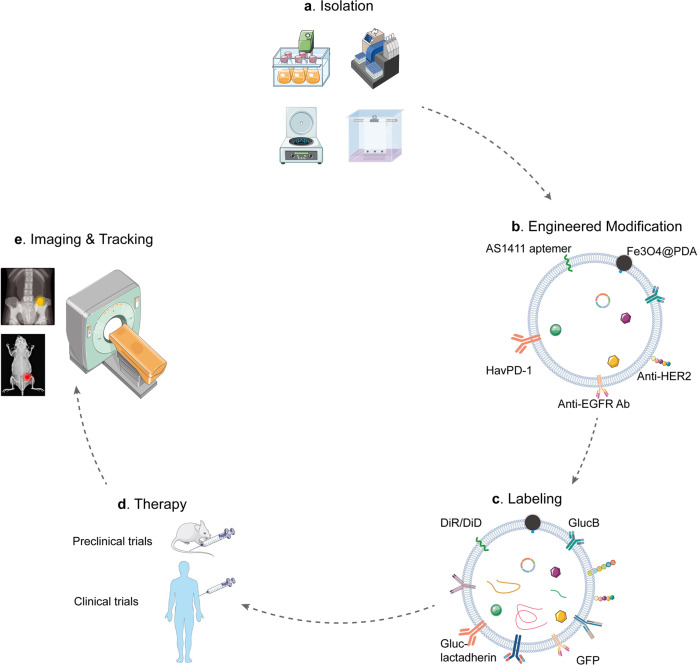
The common strategies of exosome engineering. **a** The integration of multiple basic isolation techniques is a common and efficient method to extract high-purity and high-yield exosomes. **b** The modified strategies comprise biological, immunological, physical, and chemical modification. Each strategy has its own strengths and weaknesses. Therefore, combining different modification strategies is recommended. **c** The common labeling strategies include lipid dyes (DiR/DiD), fluorescent (GFP), and bioluminescent protein (GLuc-lactadherin and GlucB). However, a ‘one size-fits-all’ reporter does not exist yet. The labeling reporter should be selected based on the practical question and available imaging equipment. **d** The common administration method of engineered exosomes is intravenous injection in both preclinical and clinical trials. **e** Based on the labeling reporter, the imaging equipment is distinct. The common tracking technique is MRI

Briefly, insolating high-purity exosomes is the first step to investigate the therapeutic functions of engineered exosomes. Conventional methods alone generally fail to meet the isolation demands, such as high purity and yield, low cost, and fewer volume limitations. The microfluidic-based isolation techniques integrate multiple pre-processing steps and methodologies in a single device, which are capable of recovering, analyzing, and quantifying exosomes within limited clinical samples, in a high-throughput manner with elevated sensitivity and multiplexing capabilities (Fig. 3a). Therefore, integrating the basic methods is essential to isolate high-purity and yield exosomes, which is the fundamental of developing efficiently engineered exosomes.

### Biological modification

Biological modification is the most widely used strategy for membrane modification, including targeted peptide and biomimetic exosome modification (Table 1). For instance, cyclo(Arg-Gly-Asp-D-Tyr-Lys) peptide (c(RGDyK)) is one of the cyclic RGD peptide (cRGD), which endows natural exosomes with a pronounced targeting ability for tumor and exerts high affinity with α_v_β_3_ integrin receptors overexpressing on the surface of tumor vascular endothelial cells or tumor cells in osteosarcoma (OS).^36–38^ Similarly, exosomes were functionalized by cyclic RGD peptide and consequently improved the specific accumulation of chemotherapeutic drugs, DOX-loaded into exosomes within tumor extracellular milieu.^39^

**Table 1:** The modification strategy and source of engineered exosomes

Modification strategy	Source	Cargo	Surface decoration	Additional stimuli	Ref.
Biological modification	Macrophage	CAT@SiO2+ ICG	AS1411 aptamer	NA	^50^
	DC	let-7 miRNA	the aptamer AS1411	NA	^201^
	3T3 and A549	anti-GFP siRNA	DOTAP, POPC, DPPC and POPG	NA	^202^
	HEK-293T	miR-155	NA	NA	^203^
	HEK-293T	miR317b-5b	NA	NA	^204^
	OS cells	lncRNA MEG3	c(RGDyK)	NA	^205^
	HEK-293T	TPD52 siRNA	DARPin G3	NA	^206^
	ASC	miR-125b	NA	NA	^207^
	4T1	antimiRNA-21 and antimiRNA-10b	uPAR	NA	^175^
	MSC	circDIDO1	RGD	NA	^208^
	HEK-293T	SIRT6 siRNA	E3-Aptamer	NA	^48^
	Normal epithelial cell	sgIQ 1.1 plasmids	HN3	NA	^209^
	Milk	β-catenin siRNA	RNA aptamers	NA	^210^
	RBC	An ASO to miR-125b	CXCR4-targeting peptide	NA	^211^
	Normal fibroblast-like mesenchymal cells	KrasG12D siRNA	anti-CD47	NA	^60^
	Dendritic cells	siGPX4	ANG	Local magnetic field	^127^
	293 T	miR-26a	Apo-A1	NA	^212^
	MDA-MB-231	miRNA-126/ miRNA-231	Integrin β4	NA	^213^
	MSC	miR-379	NA	NA	^214^
	U937	NA	anti-PSMA peptide	NA	^215^
	HEK-293	miR-HER2	NA	NA	^216^
	MSC	si-Survivin	CXCR4	NA	^217^
	HEK-293 T	CRISPR-Cas9	GFP	NA	^218^
	MSC	miR-101	NA	NA	^219^
	MSC	miRNA-29a-3p	NA	NA	^220^
	HEK293T	CRISPR-Cas9 RNP	TDNs+aptamer	NA	^221^
	M1 macrophage	NF-B p50 siRNA +miR-511-3p	IL4RPep-1	NA	^194^
	Murine muscle C_2_C_12_	Nefmut/E7	NA	NA	^222^
	M1 macrophages	NA	CD47	NA	^193^
	HEK293T	HPV-E6	NA	NA	^223^
	CAR-T	NA	EGFR and HER2	NA	^182^
	DC	NA	anti-CD3 and anti-EGFR	NA	^55^
	HEK293T	NA	rHER2/neu+ Nefmut	NA	^224^
	CT26, B16-F10, LLC, and 4T1	NA	FAP	NA	^51^
	Tumor cells nuclei-transfected M1 macrophages	NA	NA	NA	^92^
	HEK293T	NA	HPV-E7 - VLPs	NA	^225^
	B16BL6	CpG DNA	NA	NA	^86^
	MDA-MB-231	NA	HavPD-1	NA	^178^
	HEK-293T	NA	HIV-1 Nefmut	NA	^226^
	Expi293	NA	Anti-CD3 + Anti-EGFR Anti-PD-1+Anti-OX40L	NA	^179^
	J558	NA	P1A tumor antigen+ HSP70	NA	^93^
	K562	NA	HLA-A2	NA	^227^
	DC	NA	P47-P + AFP212-A2 + N1ND-N + Alarmin	NA	^183^
	K562	NA	IL-15, IL-18 and 4-1BBL (TNFSF9)	NA	^228^
	J558	TNF-a, IL-2, and IFN-g	NA	NA	^229^
	HEK293	ASO-STAT6	PTGFRN	NA	^88^
	MDA-MB-231	ELANE and Hiltonol	NA	NA	^230^
	Expi293	NA	anti-CD3 + anti-HER2	NA	^56^
Physical modification	4T1	DVDMS	NA	US	^231^
	4T1	PTX	GNR-PEG	In situ NIR	^87^
	HEK-293T	PTX + ICG + SBC	NA	US	^75^
	HEK-293T	ICG	FA	US	^232^
	HEK-293T	Ce6 + R848	NA	US	^90^
	Huh7 + MDAMB231	NIS	NA	^131^I	^130^
	M1 macrophage	DDRi, DSB	anti-PD-L1 nanobody+ CAT	Laser	^125^
	ReN	PD-L1 siRNA	RGDyK	Laser	^124^
	M1 Macrophages	ROS-sensitive Polymer+ siPAK4	NA	Laser	^141^
	HEK-293T	Er and RB	CD47	532 nm laser	^132^
	The blood of mice	NA	ChiP	Laser	^142^
	MIA-PaCa-2	Ce6	NA	Laser	^187^
	CT26	R837 + ICG	CD47	Laser	^67^
	MCF-7	V2C QDs+ TAT	RDG	Laser	^96^
	Raw 264.7	Gd and Dy-doped and TAT peptide-modified carbon dots	RGD	Laser	^163^
	4T1	Porous silicon nanoparticles+ ICG + DOX	NA	808 nm laser	^103^
	Macrophages	DOX	Fe3O4@PDA	Laser	^106^
Chemical modifiication	A549	DTX	NA	NA	^110^
	BMM	PTX	AA-PEG	NA	^111^

*CAT@SiO2* catalase into silica nanoparticles, *DTX* docetaxel, *DVDMS* sinoporphyrin sodium, *AA-PEG* aminoethylanisamide-polyethylene glycol, *PTX* paclitaxel, *BMM* bone-marrow derived macrophages, US ultrasound, *HEK-293T* human embryonic kidney-293T cells, *SBC* sodium bicarbonate, *ICG* Indocyanine green, *GNR-PEG* gold nanorods-PEG, *FA* folic acid, *ICG* Indocyanine green, *GNR-PEG* gold nanorods-PEG, *FA* folic acid, *DDRi* DNA damage repair inhibitor, *c(RGDyK)* cyclo(Arg-Gly-Asp-DTyr-Lys) peptide, *ReN cells* a neural progenitor cell line derived from the ventral mesencephalon region of the human fetal brain, *PAK4* p21-activated kinase 4, *PA* photo-activatable, *PEI* polyethyleneimine, *TK* thioketal, *Ce6* chlorin e6, *RBC* red blood cell, *ChiP* chimeric peptide, *PDA* polydopamine, *Fe3O4@PDA* polydopamine (PDA) coated magnetic Fe3O4 nanoparticles, *ASCs* adipose tissue-derived mesenchymal stromal/medicinal signaling cells, *uPAR* urokinase plasminogen activator receptor, *4T1* mouse breast cancer cells, *HN3* a human antibody targeting GPC3 with high affinity, *sgIQ 1.1* a sgRNA to direct Cas9 protein to the site of IQGAP1 in the genomic locus for efficient cleavage, *GPX4* glutathione peroxidase 4, a key regulator of ferroptosis, *siGPX4* small interfering RNA of GPX, *HDL* high-density lipoprotein, *Apo-A1* the main component of HDL, *MDA-MB-231* breast cancer, *IL4RPep-1* an IL4R-binding peptide, *HavPD-1* high-affinity variant human PD-1 protein, *J558* The myeloma cell line, *K562* the chronic myeloid leukemia cell line, *HLA-A2* human leukocyte antigen-A2, *P47-P* HCC-targeting peptide, *AFP212-A2* α-fetoprotein epitope, N1ND-N, a functional domain of high mobility group nucleosome-binding protein 1, *ASO* antisense oligonucleotide, *exoASO-STAT6* ASO targeting STAT6, *PTGFRN* prostaglandin F2 receptor negative regulator, *ELANE* human neutrophil elastase, *Hiltonol* TLR3 agonist, *MDA-MB-231* the triple-negative breast cancer cell line, *Expi293 cells* a suspension-adapted HEK293 cell line

In addition to cRGD, there are other targeted peptides that strengthen the targeting property of engineered exosomes. TLyp-1 peptide-decorated exosomes had high transfection efficiency into lung cancer for the selective target ability to neuropilin1 (NRP1) and to neuropilin2 (NRP2) of tLyp-1 peptide (amino-acid sequence CGNKRTR).^40–42^ Additionally, the exosomes could be modified with a peptide to target the mesenchymal-epithelial transition factor (c-Met) being overexpressed highly by triple-negative breast cancer (TNBC) cells.^43^ Notably, in vivo, the targeted c-Met-decorated exosomes exhibited markedly tumor-targeting efficacy.^44,45^ Therefore, modifying exosomes with peptides is a promising engineering approach.

Aptamers are short single-stranded oligonucleotides or peptides with specific three-dimensional structures, targeting cancer cells with high affinity and specificity.^46,47^ E3-aptamer-modified exosomes, carrying SIRT6 siRNA, exhibited remarkably antitumor effects on prostate cancer in vitro and in vivo.^48^ Besides, extracellular vesicles coated with RNA aptamers are capable of binding to epithelial cell adhesion molecule (EpCAM) in HCC.^49^ Similarly, AS1411 aptamer-modified macrophage exosomes present efficient blood-brain barrier (BBB) penetration and precise cancer cell-targeting capability in glioblastoma (GBM).^50^ Therefore, in addition to targeting peptide and antibody, aptamer is another direction of exosome engineering.

In brief, specific targeting peptides and aptamers are the main biological modified strategies for engineered exosomes, which endow natural exosomes with spatially targeting property (Fig. 3b).

### Immunological modification

The immunological engineered exosomes could elicit robust antitumor immunity. For example, fibroblast activation protein-α (FAP)-expressing exosomes, serving as tumor vaccine, could induce strong and specific cytotoxic T lymphocyte (CTL) immune responses against tumor cells and FAP^+^ cancer-associated fibroblasts (CAFs).^51^ Consequently, these exosome-based tumor vaccines reshaped immunosuppressive TME in colorectal cancer (CRC), melanoma, lung cancer, and breast cancer. Consistent with the above tumor vaccine, immunogenic cell death (ICD) inducers-loaded α-lactalbumin (α-LA)-decorated exosomes possessed a remarkable ability to induce ICD in a poorly immunogenic TNBC mouse xenograft model and patient-derived tumor organoids.^52–54^ In addition to tumor vaccine, anti-CD3 and anti-EGFR (epidermal growth factor receptor) antibody-engineered exosomes could promote the binding of T cells to cancer cells for precise therapy.^55^ Similarly, decorating exosomes with both anti-human CD3 and anti-human HER2 antibodies enables them to simultaneously target T-cell CD3 and breast cancer-associated HER2 receptors in HER2-expressing breast cancer.^56^ Additionally, high-affinity variant human PD-1 protein (havPD-1)-expressing exosomes could induce cellular apoptosis and efficiently block PD-L1-mediated T-cell suppression in breast cancer. Therefore, immunological engineered exosomes are a practical strategy to trigger stronger immune response inside TME.

Systemically delivered natural exosomes are easily cleared by the phagocytosis of MPS resulting in minimal accumulation within tumor, which is a major hurdle for the exosome-based therapeutic approaches. CD47, a widely expressed integrin-associated transmembrane protein that functions in part to protect cells from phagocytosis,^57,58^ is the ligand for signal regulatory protein alpha (SIRPα, also known as CD172a), and CD47–SIRPα binding initiates the ‘don’t eat me’ signal, inhibiting phagocytosis.^59^ Exosomes could be functionalized in order to improve retention time in blood with CD47 overexpression.^60–62^ Once engineered exosomes arrive in tumor sites, CD47 immune checkpoint blockade endows CRC with the macrophages-mediated phagocytosis by blocking CD47 signal.^25,63,64^

In brief, specific antibodies are the main immunological modified strategies for engineered exosomes, which endow natural exosomes with more targeting ability (Fig. 3b). The immunological engineered exosomes are expected to become a bridge connecting immunotherapy and chemotherapy with the modified surface of immune checkpoint inhibitors (ICIs) and the loaded chemotherapeutic cargos. Besides, the tumor cell-derived engineered exosomes hereditarily possess tumor-homing potential and tumor-associated antigens on surface, which could trigger a more precise and fiercer antitumor immune response inside extracellular milieu with modified targeted molecules.

### Physical modification

Engineered exosomes could achieve more accurate tumor-targeting effects with external physical interferences, such as magnetic field, laser irradiation and US (Table 1).

For example, the neutrophil-derived exosomes were decorated with superparamagnetic iron oxide nanoparticles (SPIONs), which selectively accumulate at the tumor sites under an external magnetic field.^65^ Similarly, tumor necrosis factor (TNF-alpha)-loaded and SPION-decorated exosomes could remarkably enhance cancer targeting under an external magnetic field coupled with mitigating toxicity in vitro and in vivo.^66^ In addition to magnetic targeting, external laser irradiation equally achieves high tumor-targeting therapeutic effects, especially in photothermal therapy (PTT). For instance, exosomes could be mixed with drug-loaded thermosensitive liposomes. The hybrid nanovesicles significantly target the homologous tumor in mice, remarkably accumulating at tumor sites. Notably, the photothermal agents loaded into the engineered vesicles could achieve excellent photothermal therapeutic effects under laser irradiation.^67^ Similarly, external near-infrared irradiation (NIR) could trigger the liberation of chemotherapeutic drugs controllably in glioma.^68^

However, the poor repeatability of laser irradiation extremely limits its clinical application due to radiative damage and high cost, despite the targeting drug delivery and controllable drug release. In stark contrast, given the safety and repeatability,^69^ US wave has attracted increasing interest from researchers in the field of engineered exosomes. For example, exosomes were fused with Ce6 and anchored CP05-TK-mPEG on the surface of exosomes. Under US irradiation, thioketal (TK) was broken by reactive oxygen species (ROS) produced by Ce6. Consequently, Bmp7 mRNA encapsulated into exosomes was delivered and released controllably.^28^

Taken together, the integration of internal agents and external physical interference, such as SPION with a magnetic field, thermosensor with laser irradiation, and US with Ce6, achieves not only tumor-targeting drug delivery spatially, but also drug liberation temporally (Fig. 3b).

### Chemical modification

Tumor microenvironments (TMEs) are relatively acidic probably due to the high rate of glycolysis and increased production of lactate,^70^ which is expected to contribute to the tumor-targeting modification of engineered exosomes. For example, exosomes coated with i-motifs, a cytosine-rich and pH-responsive DNA strand,^71–73^ could deliver DOX to breast cancer and release DOX in an acidic manner.^74^ Additionally, NaHCO_3_ (sodium bicarbonate, SBC) encapsulated into exosomes could generate CO_2_ bubbles rapidly after endocytosis into cancer cells, releasing paclitaxel (PTX) effectively^75^ (Fig. 3b). Except for i-motifs and SBC, a pH and temperature sensitive polymeric adhesive was developed, which can be tuned synthetically to bind to tumor cells at pH 6.8 but not at pH 7.4 at 37 °C. The pH-sensitive property enables this adhesive to serve as an essential part of engineered exosomes.^76^ Therefore, exploiting acidic TME to controllably release therapeutic cargos is a promising approach for developing a clinical-staged drug delivery system.

### The labeling and imaging of engineered exosomes

In order to capture the dynamics of physiological and pathophysiological activities of exosomes, appropriate imaging strategies are essential to monitor engineered exosomes in vivo, such as exosomes labeling strategies, reporter systems, and microscopy techniques.

Labeling strategies are necessary for imaging engineered exosomes in vivo. Lipid dyes, luminal dyes, and genetic labeling are the main strategies to label engineered exosomes. For example, PKH67, DiR/DiD, and MemGlow, as lipid dyes, have been used to label engineered exosomes.^77^ However, as the half-life of lipid dyes greatly exceeds that of engineered exosomes,^78,79^ exosomes degradation after cellular uptake is masked by recycling and redistribution of fluorescent dye. Additionally, the investigations of therapeutic effects of engineered exosomes on tumors are long-term studies. Therefore, lipophilic dye labeling may not be best suited for cancer studies. Apart from lipophilic dye, a number of genetically encoded reporters have been developed to label engineered exosomes using fluorescence or bioluminescence. Labeled proteins in cytosol can be loaded into the lumen of engineered exosomes.^80^ Besides, reporters (including GFPs, RFPs, and the bioluminescent ThermoLuc) are attached to engineered exosomes cargos, such as TSPAN4, CD63, CD81, and CD9, of which CD63 is the most widely used.^15–17,81,82^ Compared with fluorescent proteins, bioluminescent proteins emit signal after substrate addition with a high signal-to-noise ratio but comparatively lower spatiotemporal resolution.^83^ Therefore, bioluminescence-based reporters, such as gLuc-lactadherin and GlucB are recommended for labeling engineered exosomes in small animal models at whole-animal.^84,85^ The administration of engineered exosomes is equally essential. In most in vivo experiments, mice with tumors were treated with intravenous injections (tail vein). In some other researches, the mice received intratumoral,^86–90^ intraperitoneal^60,91^ or intradermal^92,93^ injection of engineered exosomes. Additionally, intestinal veins (ISVs) injection was reported to be used in zebrafish model.^94^ In clinical trials, engineered exosomes might be administrated via intradermal injections. For example, in one phase II trial, the patients with unresectable NSCLC received tumor antigen-loaded dendritic cell-derived exosomes (Dex) with intradermal injections (NCT01159288).

In addition to successful labeling, live imaging of engineered exosomes is also significant in vivo studies. There are various alternative microscopies for exosomes researches, such as stochastic optical reconstruction microscopy (STORM), structured illumination microscopy (SIM), and Confocal laser scanning microscopy (CLSM). SIM and CLSM can detect engineered exosomes in the sub-200-nm range, tracking their uptake by living cells, and their dynamic intracellular distribution on a time scale of seconds, which are suitable to investigate the physiological and tumoricidal effects of engineered exosomes on cancer cells. In some experiments, given the availability of imaging equipment, the researchers also used MRI to detect the biological action of engineered exosomes in vivo.^95,96^

Taken together, the integration of bioluminescent proteins and SIM or CLSM is one appropriate strategy for live imaging of engineered exosomes in vivo. However, there is no doubt that a ‘one-size-fits-all’ reporter and microscopy does not exist yet, and a particular combination of reporter and microscopy is supposed to be chosen based on the biological question and practical imaging equipment (Fig. 3e).

## Engineered exosomes, an ideal executor for clinical therapeutic modalities

### Chemotherapy

Systemic administration of chemotherapeutic drugs generally leads to various TRAEs, including peripheral sensory neuropathy,^97,98^ myelosuppression^98,99^ and cardiac toxicity.^100^ Beyond these TRAEs, the reduced antitumor efficiency is another hurdle for the elongation of survival periods, due to the lack of targeting ability of chemotherapeutic drugs. Given the limitations above, it is now the time for engineered exosomes to make their way into history for their tumor-targeting competence (Table 2). For example, DOX, a kind of broad-spectrum chemotherapeutic drug, could be encapsulated into various exosomes from distinct cells.^39,43,65,101–108^ The DOX-loaded and targeted molecules-decorated exosomes achieve pronounced antitumor effects with less major organs damage^65,101,104–106,109^ in preclinical models, including heart, liver, spleen, lung, and kidney. Particularly, a kind of NIR light-responsive macrophage-derived exosomes were designed, composing of DOX and 4.2 nm Ag_2_S quantum dots (QDs). The “Trojan horse” could be monitored by NIR fluorescence imaging of the Ag_2_S QDs and release DOX under the irradiation of NIR. Most surprisingly, the QDs and DOX can synergistically penetrate the whole tumor with a diameter of about 9 mm, which is superior to DOX administration alone.^109^

**Table 2:** The preclinical trials of engineered exosomes

Tumor models	Putative name	Clinical therapy	Mechanisms	Ref.
NSCLC	AA-PEG-exoPTX	CT	Target the sigma receptor on lung cancer cells	^111^
NSCLC	EXO-DTX	CT	Enhance the cytotoxicity of DTX	^110^
BC	SBC-EV(ICG/PTX)	CT	Improve the cellular uptake of ICG Release the PTX in response to acidic pH in the endo/lysosomes	^75^
BC	TEX-Liposome-PTX-GNR-PEG	CT	Activate GNR-mediated thermal ablation Increase the level of CD8+ T cells in lungs Improve the concentration of serum cytokines (tumor necrosis factor-α, interlekin-6, and interferon-γ)	^87^
BC	DOX -TEVGIONs	CT	Block the function of endogenous oncogenic miR-21 Attenuate DOX resistance Enhance T2 contrast in in vitro MR imaging	^95^
CRC	THLGEXO/5-FU/miR-21i	CT	Induce cell cycle arrest, reduced tumor proliferation, increased apoptosis and rescued PTEN and hMSH2 expressions Reverse drug resistance and significantly enhanced the cytotoxicity in 5-FU-resistant colon cancer	^233^
CRC	PGM5‐AS1-oxaliplatin-EXO	CT	Prevent proliferation, migration, and acquired oxaliplatin tolerance Reverse drug resistance Activate alternate splicing to downregulate PAEP expression Act as a sponge to upregulate the NME1 expression	^89^
LC	DDRi@CAT-PD-M1Exos	RT	Relieve tumor hypoxia Enhance DNA damage Inhibit DNA damage repair Polarize M2 macrophages into M1 phenotypes Relieve the immunosuppression of T cells	^125^
HCC	NIS-EV	RT	Facilitate radioiodine uptake Enhance the antitumor effects of ^131^I radiotherapy	^130^
GBM	RGD-siPD-L1-EV	RT	Enhance the targeting efficiency of RGD-EV Reverse radiation-stimulated PD-L1 expression Recruit tumor-associated myeloid cells Increase CD8+ cytotoxic T cells	^124^
Melanoma/CRC	PASEV	PDT	Release ROS-responsive siPAK4 Prime the TME Boost intratumoral infiltration and immune activation	^141^
HCC	Er/RB@Exos^CD47^	PDT	Make the exosomes effectively escape the phagocytosis of MPS Induce ferroptosis after irradiation of 532 nm laser	^132^
CRC/BC	ChiP-Exo	PDT	Disrupt the membrane integrity Improve the cytosolic delivery of ChiP-Exo under the first-stage light Enhance its nuclear delivery under the second-stage light Activate ROS in situ to disrupt nuclei	^142^
Pancreatic cancer	Ce6-R-Exo	PDT	Generate ROS inside tumor cells under laser irradiation Increase the release of cytokines from immune cells	^187^
CRC	I/R@hGLV	PTT	Increase the long blood circulation Improve the macrophages-mediated the phagocytosis of tumor cells Lead to immunogenic cell death Generate TAA Promote the maturation of immature DCs	^67^
BC	TEX-Liposome-PTX	PTT	Activate the adaptive antitumor immune response Increase the level of CD8+ T cells Improve the concentration of serum cytokines Enhance The therapeutic efficacy combined with thermal ablation, adaptive antitumor immunotherapy, and targeted PTX chemotherapy.	^87^
BC/ LC	V2C-TAT@Ex-RGD	PTT	Target the cells Enter the nucleus to realize low-temperature PTT	^96^
BC/ CC	CDs:Gd,Dy-TAT@Exo-RGD	PTT	Accumulate at cancer site with an increased concentration CDs induce localized hyperpyrexia to ablate tumors Exhibit higher MRI/CT imaging contrast enhancement of tumor sites	^163^
BC	ID@E-MSNs	PTT	Retain the photothermal effect of ICG and cytotoxicity of DOX Produce hyperthermia to collapse E-MSNs nanovehicles, accelerate drug release, and induce tumor ablation under 808 nm near-infrared irradiation Inhibit the growth and metastasis of tumor	^103^
BC/CC/leukemia	Exo-DOX-Fe3O4@PDA-MB	PTT	Enable enrichment of the exosomes at the tumor site by external magnetic field guidance. Utilize localized hyperthermia to trigger the release of cargoes Target the miR-21 for both imaging and gene silencing Kill the cancer cells via DOX	^106^
GBM	CAT@SiO2-ICG	SDT	Possess efficient BBB penetration and good cancer cell-targeting capability Relieve tumor hypoxia	^50^
PC	Exo^Ce6+R848^	SDT	Enhanced R848-mediated DCs maturation Reprogram macrophages from M2 phenotype to M1 phenotype Activate effector T cells Revert the immunosuppressive TME	^90^
BC/CRC	EXO-DVDMS	SDT	Trigger DVDMS relocation Initiate multiple cells death-signaling pathways Facilitate simultaneous imaging and tumor metastasis inhibition	^231^
BC	SBC-EV(ICG/PTX)	SDT	Improve the cellular uptake of ICG Release the PTX in response to acidic pH in the endo/lysosomes Burst exosome membranes by CO2 bubbles	^75^
BC	FA-ExoICG	SDT	Improve aqueous stability Promote cellular uptake of ICG Increase ROS generation Trigger sonotoxicity against cancer cells	^232^
BC	Let-7 miRNA -AS1411-Exo/VEGF siRNA -AS1411-Exo	GT	Inhibit malignant growth of cancer cells by reducing MYC and RAS expression	^201^
BC	HER2/Neu-siRNA TPD52-Exo	GT	Bind specifically to HER2/Neu Deliver siRNA molecules against TPD52 gene	^206^
BC	miR-379 -Exo	GT	Elevate miR-379 Reduce COX-2 mRNA and protein in vitro and in vivo	^214^
BC	uPA-eEV-PNCs	GT	Overexpress miRNA-10b and miRNA-21 Alleviate chemoresistance and metastatic potential	^175^
BC	293-miR-XS-HER2	GT	Block HER2 synthesis Adhere to HER2 on the surface of cancer cells	^216^
BC/ Leukemia	ASO^CXCR4+EGFR+EpCAM^ -Exo	GT	Knockdown oncogenic miR-125b Target to CXCR4	^211^
BC/l LC	miRNA-231-Exo	GT	Recognize lung cancer cells in blood Escape from the immune surveillance system in vitro Suppress lung cancer cell proliferation and migration Interrupt the PTEN/PI3K/AKT pathway	^213^
BC/HCC/CC	CRISPR-Cas9-Exo	GT	Downregulate GFP or WNT10B Reduce WNT10B in vitro, ex vivo, and in vivo	^221^
OS	miR-317b-5b-Exo	GT	Enhance the internalization of miR317b-5b in tumor cells Suppress cell viability, proliferation, migratory and invasive capability Promote apoptosis	^204^
OS	cRGD-Exo-MEG3	GT	Unclear	^205^
OS	miR-101-Exo	GT	Reduce BCL6 mRNA and protein Target BCL6 via miR-101	^219^
LC	Hybrid Lipid-Exo	GT	NA	^202^
LC	β-catenin siRNA-Exo	GT	Bind to EpCAM Decrease β-catenin expression and proliferation	^210^
HCC	miR-26a-Exo	GT	Bind selectively to cancer cells via the scavenger receptor class B type 1–Apo-A1 complex Promote the internalization by receptor-mediated endocytosis Upregulate miR-26a expression Decrease the rates of cell migration and proliferation	^212^
HCC	HN3LC9-293exo	GT	Target GPC3 with high affinity Direct Cas9 protein to the site of IQGAP1 in the genomic locus for efficient cleavage	^209^
HCC	miR-125b-Exo	GT	Overexpress miR-125b in HCC cells Induce cell cycle arrest Inhibit proliferation, migration, and invasion	^207^
GC/CRC/ LC	CXCR4^high^ Exo/si-Survivin	GT	Bind to the highly expressed stromal cell-derived factor-1 (SDF-1) on the tumor surface Knock down the Survivin gene in tumor cells in vivo and thereby inhibiting tumor growth	^217^
PC	PSMA-EMs	GT	Trigger PSMA-mediated endocytosis Release drug intracellularly	^215^
PC	SIRT6 siRNA-Exo	GT	Activate multiple cancer-related signaling pathways, especially the Notch pathway Silence SIRT6 Inhibit tumor growth and metastasis	^48^
LC	sgRNA:Cas9-Exo	GT	Enrich sgRNAs and Cas9 proteins in exosomes using GFP-binding nanobody	^218^
PDAC	Kras^G12D^ RNAi-Exo	GT	Knockdown KrasG12D via siRNA or shRNA	^60^
GBM	MNP@BQR@ANG-EXOsiGPX4	GT	Accumulate in the brain under local magnetic localization Trigger transcytosis Target GBM cells by recognizing the LRP-1 receptor Trigger ferroptosis by the combined triple actions of the disintegration of dihydroorotate dehydrogenase and the glutathione peroxidase 4 ferroptosis defense axis with Fe_3_O_4_ nanoparticle-mediated Fe^2+^ release	^127^
GC	RGD-Exo-circDIDO1	GT	Inhibit GC progression by regulating the expression of the signal transducer inhibitor SOSC2 through sponging miR-1307-3p	^208^
Glioma	miR-29a-3p-Exo	GT	Inhibit migration and VM formation Target ROBO1 via miR-29a-3p	^220^
BC	IL4R-Exo(si/mi)	IT	Foster M1 polarization by NF-B p50 siRNA and miR-511-3p Target the IL4R of TAMs	^194^
BC	aCD47/aSIRPα-Exo	IT	Cleave the benzoic-imine bonds of exosomes nanobioconjugates in the acidic TME Block SIRPα on macrophages and CD47 Transit the macrophages from pro-tumoral M2 to antitumoral M1	^193^
BC	GEMINI-Exos	IT	Redirect and activate T cells toward killing EGFR-positive TNBC	^179^
BC	HPV-E7-Exo	IT	Trigger a stronger antigen cross-presentation in both B- lymphoblastoid cell and monocyte-derived immature DCs Increase TAA-specific CD8+ T cell	^225^
BC	HELA-Exos	IT	Induce ICD in breast cancer. Activate cDC1s in situ Cross-prime tumor-reactive CD8+ T-cell response	^230^
BC	SMART-Exos	IT	Target T-cell CD3 and breast cancer-associated HER2 receptors dually Redirect and activate cytotoxic T cells toward attacking HER2-expressing breast cancer	^56^
BC/NSCLC	EGFR / HER2 CAR -Exo	IT	Express a high level of cytotoxic molecules without PD1	^182^
BC/ LC	HER2/neu/Nefmut -Exo	IT	Target antigen-specific CD8+ T lymphocytes Activate HER2-directed CTL activity	^224^
BC/Lymphoma/Melanoma	aMT-exos	IT	Prime T-cell activation in both the classical antigen-presenting cell-induced immunostimulatory manner and a unique “direct exosomes interaction” manner Ameliorate immunosuppression	^92^
BC/CRC/NSCLC/Melanoma	eNVs-FAP	IT	Induce strong and specific CTL immune responses against tumor cells and FAP + CAFs Reprogram the immunosuppressive TME Promote tumor ferroptosis by releasing interferon-gamma (IFN- γ) from CTLs and depleting FAP + CAFs.	^51^
Melanoma	CpG-SAV-exo	IT	Activate DC2.4 cells Enhance tumor antigen presentation capacity Exhibit stronger antitumor effects in vivo	^86^
Melanoma	Exo-OVA-aCD3/aEGFR	IT	Activate endogenous T cells efficiently Crosslink with cancer cells Upregulate PD-L1 expression	^55^
LC	Nef^mut^/E7-Exo	IT	Trigger CD8+ T-cell immune response	^222^
B-LCL	HPV-E6-Exo	IT	Trigger a stronger antigen cross-presentation in both B- lymphoblastoid and monocyte-derived immature DCs Increase TAA-specific CD8+ T cells	^223^
Myeloma	EXO_HSP_	IT	Stimulate maturation of DCs Activate Th1 cell responses, and more efficient P1A-specific CD8+ CTL responses	^93^
CML	CoEX-A2s	IT	Stimulate antigen-specific CD8+ T cells both directly and indirectly via CoEX-A2 crossdressed cells Generate HCMV pp65-specific and MART1-specific CD8+ T cells as DEX in vitro	^227^
HCC	DEXP&A2&N	IT	Promote recruitment, accumulation and activation of DCs Enhance cross-presentation of tumor neoantigens and de novo T-cell response. Increase immunological memory against tumor re-challenge	^183^
Myeloma	EXO_TNF-a_	IT	Induce more efficient P1A-specific CD8þ T-cell response	^229^
CRC	ExoASO-STAT6	IT	Silence STAT6 expression in TAMs Remodel the TME Generate CD8+ T cell-mediated adaptive immune response	^88^

*CC* cervical cancer, *OS* osteosarcoma, *PC* prostate cancer, *GBM* glioblastoma, *PDAC* pancreatic ductal adenocarcinoma, GC gastric cancer, *BC* breast cancer, *NSCLC* Non-small cell lung cancer, *LC* lung cancer, *CML* chronic myelogenous leukemia, *HCC* hepatocellular cancer, CRC Colorectal cancer, *STAT6* signal transducer and activator of transcription 6, *IT* immune therapy, *CTL* cytotoxic T, lymphocyte, ICD immunogenic cell death, *TNBC* triple-negative breast cancer, *TAMs* tumor-associated macrophages, *GBM* glioblastoma, *GFP* green fluorescent protein, *PSMA* prostate-specific membrane antigen, *GT* gene therapy, *BCL6* B cell lymphoma, *CXCR4* CXC chemokine receptor type 4, *HER2* human epidermal growth factor receptor 2, *ICG* Indocyanine green, *SDT* Sonodynamic therapy, *BBB* blood-brain barrier, *DCs* dendritic cells, *TAA* tumor-associated antigen, *PTT* photothermal therapy, *PDT* Photodynamic therapy, *ROS* reactive oxygen species, *TME* tumor microenvironment, *RT* radiotherapy, *5-FU* 5-Fluorouracil, *MR* magnetic resonance, *GNR* gold nanorods, *PTX* paclitaxel, *DTX* docetaxel, *CT* Chemotherapy, *GNP* gold nanoparticle, *SIRPα* signal regulatory protein α, *DOX* doxorubicin

Besides DOX, docetaxel, and PTX can also trigger more efficient antitumor response after being encapsulated into exosomes. For instance, in breast cancer, PTX-loaded exosomes not only inhibit tumor growth,^75^ but also prevent recurrence and metastasis in breast cancer-bearing mice.^87^ More importantly, PTX release is enhanced by both SBC and US irradiation, demonstrating dual pH/US-responsive drug release.^75^ However, the two studies exploited the HEK-293T cells and tumor-derived exosomes without specific targeting decoration, which end up with a relatively high proportion of drug-loaded exosomes accumulating in liver and spleen, the major organs enriching MPS. In lung cancer, there are also reports about engineered exosomes loaded with docetaxel^110^ and PTX,^111^ resulting in encouraging antitumor response in preclinical models. Engineering exosomes from macrophage with incorporated aminoethylanisamide-polyethylene glycol (AA-PEG) and PTX had pronounced anticancer impact on a mouse model of pulmonary metastases.^111^ PEG reduces the recognition and internalization by MPS and therefore avoids being cleaned, significantly increasing the circulation time of engineered exosomes in vivo.^112^

Collectively, chemotherapeutic drug-loaded exosomes could elicit more powerful antitumor activity with less systemic toxicity than that chemotherapy alone. Meanwhile, proper modification of the exosomes’ membrane endows them with enhanced tumor-targeting and intracellular delivery capabilities.

### Radiotherapy

Radiotherapy has a key role across the disease spectrum in nearly every solid cancers, including glioblastoma, cervical carcinoma, lung cancer, and breast cancer^113^ (Table 2). Radiotherapy is proven to cause satisfying outcomes, with or without other therapies, such as surgery, targeted therapy, chemotherapy, and immunotherapy.^114–116^ Despite the wide clinical application and satisfying outcomes, there are still some intrinsic hurdles limiting its efficacy. For example, radio-resistance inherent in various cancers (e.g., CRC and lung cancer) leads to poor response of multiple malignant patients initially.^117–121^ Besides, owing to physical barriers, such as BBB, major therapeutic molecules fail to exert antitumor effects with irradiation spontaneously in GBM.^122,123^ Given the above limitation, it is time for engineered exosomes to make their way into history. The combination of radiotherapy and engineered exosomes with inherent surface proteins and synthetic targeting molecules, such as brain-tumor-targeting cyclic RGDyK peptide,^124^ not only alleviates radio-resistance and subsequently reboot immune response, but also increases the accumulation of drug-loaded exosomes in tumor sites.

One example of radiotherapy combined with engineered exosomes is that M1 macrophage-derived engineered exosomes (M1Exos) were developed in order to sensitize Lewis lung carcinoma-bearing mice to radiotherapy.^125^ Mechanistically, catalases expressed on the inside of M1Exos membrane could effectively alleviate hypoxia inside TME and strengthen DNA damage synchronously. Additionally, the DNA damage repair inhibitor previously encapsulated into M1Exos could limit DNA damage repair significantly.^125^ Furthermore, M1Exos have largely been shown to polarize macrophage from M2 to M1 phenotypes. Finally, the synthetic anti-PD-L1 nanobody on the surface of M1Exos was found to terminate the immunosuppression of T cells. Owing to above modifications, engineered M1Exos solved three limitations that compromise the efficacy of radiotherapy through the relief of tumor hypoxia, the inhibition of DNA damage repair, and the remodeling of immunosuppressive TME.

Another example of radiotherapy combined with engineered exosomes is that the bursts of radiation could mediate the penetration of engineered exosomes through BBB and ultimately increase the accumulation of exosomes inside GBM.^124^ Three main mechanisms have been presented to facilitate the penetration of exosomes into the brain by crossing the BBB: invasive, pharmacological, and physiological approaches.^126^ Invasive approaches deliver the active compounds by breaching the BBB. For example, laser^124^ and ultrasound radiation^50^ might compromise the integrity of BBB, providing engineered exosomes with window to cross BBB. The pharmacological approach is based on the modification of active compounds and/or their formulation in order to give them attributes allowing their entry into the brain passively. For instance, engineered exosomes with magnetic nanoparticles were proved to cross BBB more efficiently under a magnetic field than the control.^65,127^ The physiological approach benefits from the occurrence of endogenous receptors that are highly expressed at the surface of the BBB, for example, angiopep-2^127^ and CD63.^128^ In addition to the above receptors, inflammatory factors released after tumor resection guide the passage of the engineered exosomes cross BBB.^129^ To conclude, the physiological approach seemed to be the most efficient strategy for promoting engineered exosomes cross BBB in neurological tumor.

In addition to external beam radiotherapy (EBRT), the efficacy of ^131^I internal radiotherapy could also be augmented by the sodium iodide symporter (NIS) protein-loaded exosomes. The NIS-loaded exosomes increase the amount of NIS protein on the plasma membrane of liver cancer cells, which facilitate radioiodine uptake and consequently promotes ^131^I internal radiotherapy.^130^ The results proved significantly greater antitumor effect of ^131^I radionuclide therapy following exosome NIS delivery. This supports the potential clinical application of exosomes as a NIS protein delivery system for cancer therapy.^130^ However, the study was not conducted in vivo, which impairs the reliability of the research results.

To sum up, the radio-resistance and physical barriers indeed constrain the efficacy of radiotherapy alone or with other therapies. However, engineered exosomes with proper modifications, such as surface engineering, drug loading, and other therapeutic combination, shed the light on the enhanced therapeutic outcomes of radiotherapy at the same dose of irradiation.

### Photodynamic therapy

Photodynamic therapy (PDT) is a non/minimally invasive cancer treatment approach, exploiting the photosensitizer (PS)-generated ROS to treat cancers^131^ (Table 2). Engineered exosome-based PDT is mainly classified into three categories: (1) the laser irradiation generates ROS, inducing ferroptosis in tumor cells, (2) the laser irradiation initiates ROS-responsive release of therapeutic molecules spatially and temporally, (3) dual-stage light triggers plasma membrane rupture and nucleus damage.^132^ Activating ferroptosis inducer and photosensitizer carried by exosomes via laser irradiation could achieve synergetic antitumor effects.^132,133^ Mounting evidence has shown ferroptosis could exhibit a pronounced therapeutic impact on plenty of solid tumors, such as gastric cancer, pancreatic cancer, CRC, and HCC particularly on apoptosis-resistant tumors.^134–136^ Ferroptosis relies on iron-dependent Fenton reaction and the accumulation of lipid peroxides.^137^ One of the characteristics of ferroptosis is the accumulation of lipid ROS. Therefore, delivering Rose Bengal (a photosensitizer)^138^ and Erastin (a classic ferroptosis inducer)^137^ to tumor sites via engineered exosomes leads to synergistic efficient ferroptosis induction with less severe side effects in HCC mice model.^132^ In addition to inducing ferroptosis, PDT serves to release therapeutic molecules encapsulated into exosomes controllably. For example, p21-activated kinase 4 (PAK4) is a significant target for tumors to prevent intratumoral immune infiltration.^139,140^ The siRNAs against PAK4 (siPAK4s) were designed and assembled with a photo-activatable ROS-sensitive polymer. After that, this nanocomplex core was camouflaged by exosomes from M1 macrophages. Under laser irradiation, these engineered exosomes not only silenced PAK4, but also primed TME through PDT, hence synchronously boosting intratumoral infiltration and immune activation.^141^ This research illustrated a new avenue to sensitize cancer immunotherapy that may be applied to treat other cancers for superior antitumor efficacy.

Another example of engineered exosome-based PDT is the dual-stage light-guided plasma membrane and nucleus-targeting photodynamic therapy.^133,142^ Under the first-stage light, multifunctional chimeric peptide-engineered exosomes could directly disrupt the membrane structure and lead to cell death. Meanwhile, photochemical internalization (PCI) effects^143^ and lysosomal escape^142^ resulting from first-stage light would improve the cytosolic delivery of exosomes. Guided by the nuclear localization signals (NLS) peptide,^144^ exosomes would penetrate nuclei effectively. Subsequently, intranuclear exosomes could generate ROS in situ to destroy nuclei for enhanced synergetic PDT under the second-stage light. Compared to conventional PDT, dual-stage PDT shows a remarkably enhanced effect on the inhibition of tumor development without serious systemic toxicity. A multifunctional chimeric peptide (ChiP), C16-K(PpIX)-PKKKRKV, consists of an alkyl chain, a photosensitizer of PpIX and a NLS peptide for surface adhesion, PDT, and nuclear translocation respectively.^142^ Notably, the endo/lysosomal entrapment and enzymatic degradation is a severe hurdle, despite the link of photosensitizer to NLS peptide promoting its nuclear penetration efficiency.^144,145^

Collectively, photosensitizer and ferroptosis inducer loaded in engineered exosomes could elicit synthetic antitumor effects. Additionally, ROS from PDT could serve as a key to release the therapeutic molecules in specific temporal and spatial manners to alleviate TRAEs, which is the greatest progress compared to the traditional systemic administration. Furthermore, the dual-stage PDT could generate ROS and kill cancer cells selectively and sequentially.

### Photothermal therapy

Compared to PDT generating ROS, photothermal therapy (PTT) exploits hyperthermia, generated by photothermal agents (PTAs), upon NIR laser radiation, to ablate tumor cells^146^ and liberate drug controllably^147,148^ (Table 2). ICG is one of the common classical PTAs, which could trigger apoptosis in tumor under laser irradiation.^149,150^ However, because of the hydrophobic characteristic of ICG,^151^ it is necessary to encapsulate ICG into a drug delivery platform to improve the solubility of ICG. Therefore, a combinatorial treatment platform was presented, which integrated CD47-overexpressed exosomes and ICG to resolve the above problem and consequently enhance the efficacy of ICG-mediated PTT.^67,152^ Additionally, another drug delivery system was designed based on exosomes-camouflaged porous silicon nanoparticles, to co-load ICG and DOX. These engineered exosomes not only elicit synergistic antitumor effects of chemotherapy and PTT, but also address the hydrophobicity of ICG and the toxicity of DOX in vivo.^103^ Similar to ICG, polydopamine (PDA) is another common PTA, which could convert NIR irradiation into heat energy.^153,154^ Therefore, exosomes-mediated multi-mode therapeutic platform was developed, composing of PDA on the surface, DOX, and miR-21 in the cavity. The heat energy caused by NIR light leads to the instability of exosomes, hence accelerating the releasing of DOX and miR-21.^106^

However, the tumoricidal efficacy of PTT generally is impaired by the limited penetration depth in the NIR-I biowindow and the heat shock protein (HSP)-mediated thermoresistance.^155,156^ Additionally, in order to ablate tumor tissue efficiently and overcome the HSP-caused thermoresistance, a high temperature of over 50 °C is essential, which inevitably leads to heating damage of normal organs near the tumor.^157^ Alternatively, the second NIR (NIR-II) biowindow (1000−1350 nm) exhibited deeper tissue penetration than that of the NIR-I biowindow (750−1000 nm).^158^ Particularly, ultrasmall 2D vanadium carbide quantum dots (V2C QDs) possess intense photothermal effects at the NIR-II biowindow. Researchers conjugated V2C QDs with nucleus-targeting TAT peptides (V2C-TAT),^159^ which served as therapeutic core nanoparticles and were loaded into Arg-Gly-Asp (RDG) peptide-decorated exosomes. These modifications endow the engineered exosomes with the cancer cell membrane and nucleus organelle dual-targeted capability. In tumor-bearing mice, the engineered exosomes present potent antitumor effects.

In addition to vanadium carbide, another mental nanomaterial, rare earth elements Gd and Dy could mediate imaging-guided PTT. Researchers linked RGD and TAT, a plasma membrane-targeting peptide and nucleus-targeting peptide respectively, on the exosomes. Furthermore, Gd and Dy-doped carbon dots (CDs) were generated and coated by PDA.^160–162^ Eventually, they encapsulated CDs into exosomes. In tumor-bearing mice, these engineered exosomes accumulate at tumor sites under the Gd and Dy-mediated images and exert pronounced antitumor effects.^163^

In brief, compared with ICG-based conventional PTT, the biomimetic nanoparticle-based dual-targeted or dual-stage PTT possesses more advantages. The dual-targeted or dual-stage PTT not only overcomes the HSP-mediated thermoresistance, but also enhances the penetration depth of PTT, which decrease the dose of laser. Furthermore, combining chemotherapy and PTT could improve the susceptibility of chemotherapeutic agents, reduce the drug dose and overcome multi-drug resistance. It is envisaged that developing more advanced biomimetic nanomaterials, in particular mental nanoparticles, could remarkably expand the application of PTT.

### Sonodynamic therapy

Sonodynamic therapy (SDT), a kind of ROS-mediated cancer therapy approach, utilizes US and sonosensitizers to kill tumors^164^ (Table 2). Unlike ROS-mediated PDT, which is limited by the penetration depth and damage to normal tissue, SDT could treat deep-seated tumors safely with sonosensitizer-loaded exosomes.^164^ For example, owing to the BBB and hypoxic TME, conventional SDT generally leads to poor outcomes.^165,166^ However, AS1411 aptamer-modified macrophage exosomes possess efficient BBB penetration and precise cancer cell-targeting capability,^167^ which consists of catalase (CAT)-loaded silica nanoparticles (CAT@SiO2) and the sonosensitizer ICG. Besides, the oxygen from CAT-catalyzed endogenous hydrogen peroxide could efficiently overcome tumor hypoxia.^168^ In vivo experiments demonstrated that these engineered exosomes promoted the efficacy of SDT, which showed a promising perspective for clinical translation.

Engineered exosome-based SDT not only possesses impotent antitumor property, but also exerts synergistic effects while combing with other therapies. Mounting evidence showed that engineered exosome-based SDT, combining with other therapies, could achieve synergistic effects. For example, dual stimuli-responsive sonosensitizers were designed, which possessed desirable biosafety using exosomes. ICG, approved by FDA as a sonosensitizer and photoacoustic (PA) imaging agent, was encapsulated into exosomes, coupled with PTX and SBC, achieving pH-responsive PA imaging-guided chemo-sonodynamic combination therapy. SBC-EVs(ICG/PTX) are accumulated at tumor sites under the vision of high-resolution PA imaging in breast tumor-bearing mice. It is worth noting that systemic administration of SBC-EV(ICG/PTX) followed by US irradiation markedly suppressed tumor growth in mice without systemic toxicity.^75^ The dual pH- and US-responsive SBC-loaded EVs regulated the release of PTX more precisely at the tumor sites, which promoted high anticancer effects of chemo-SDT in target cancer cells.

Briefly, engineered exosomes combining with US irradiation not only show high biocompatibility, long blood retention, and precise tumor targeting, but also achieve drug release controllably and no adjacent normal tissue damage. These combination therapies improve the sensation of antitumor therapeutics, therefore reducing the dose of administration and TRAEs. Looking forward, engineered exosome-mediated sonodynamic therapy has a promising perspective for clinical translation.

### Gene therapy

**Fig. 4:**
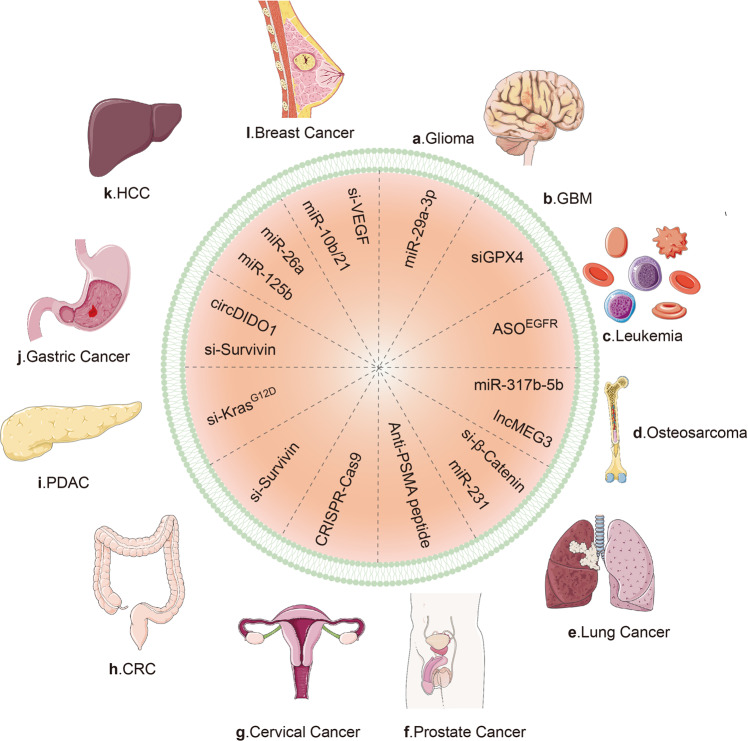
Genetically engineered exosomes in various cancers. **a** In glioma, miR-29a-3p-loaded exosomes inhibit tumor migration and vasculogenic mimicry by targeting ROBO1. **b** In glioblastoma, MNP@BQR@ANG-EXOsiGPX4 platform delivers siGPX4 into tumor cells and subsequently knockdown GPX4, inducing stronger lipid peroxidation and ferroptosis. **c** In leukemia, ASO^EGFR^ efficiently target EGFR on the cancer cells. **d** In osteosarcoma, the internalization of miR317b-5b resulted in changes of tumor progression. And cRGD-Exo-MEG3 achieved stronger anti-OS effects than lncMEG3 alone. **e** In lung cancer, miRNA-231-Exo strongly inhibited cancer proliferation and migration by interrupting the PTEN/PI3K/AKT signaling pathway. Meanwhile, si-β-Catenin led to loss of β-catenin expression and decreased proliferation. **f** In prostate cancer, anti-PSMA peptide bind to PSMA receptor on the PC cells, triggering receptor-mediated endocytosis and thereafter intracellular drug release. **g** In cervical cancer, the exosome-based CRISPR-Cas9 delivery exhibited significant cancer suppression. **h** In colorectal cancer, si-Survivin knocked down the Survivin gene in tumor cells in vivo. **i** In PDAC, si-Kras^G12D^ suppressed cancer in multiple mouse models of pancreatic cancer and significantly increased overall survival. **j** In gastric cancer, CircDIDO1 regulated the level of the signal transducer inhibitor SOSC2 through sponging miR-1307-3p, therefore inhibiting GC progression. **k** In hepatocellular carcinoma, miR-26a decreased cell migration and proliferation. **l** In breast cancer, si-VEGF loaded into exosomes exhibited more significant antitumor effects than si-VEGF alone. PC prostate cancer. CRC colorectal cancer. PDAC pancreatic ductal adenocarcinoma. PSMA prostate-specific membrane antigen. GPX4 glutathione peroxidase 4. ASO antisense oligo nucleotide. exoASO-STAT6 ASO targeting STAT6. EGFR epidermal growth factor receptor. cRGD-Exo-MEG3 c(RGDyK)-modified and MEG3-loaded exosomes. LncRNA MEG3 long non-coding RNA maternally expressed gene 3

Gene therapy is the transfer of genetic molecules to a patient, so as to treat a disease via durable expression of the therapeutic gene or “transgene” to alleviate or even cure disease symptoms without severe adverse events^169,170^ (Table 2). Non-coding RNA (ncRNA) can serve as the vector of gene therapy in multiple diseases, including cancers.^171–174^ However, with respect to the level, timing, and location of gene therapy, there still exist huge challenges in the controllable expression of ncRNA therapeutics, which is a major hurdle for ncRNA-based therapies. Fortunately, advances in engineered exosomes provide the hope that it is realistic to deliver therapeutic ncRNAs to tumor sites in precise temporal, spatial, and dose manners by loading ncRNAs into engineered exosomes (Fig. 4). For example, antimiRNA-21 and antimiRNA-10b were loaded into polymeric nanocarriers (PNCs) and subsequently coat these PNCs with uPA engineered exosomes (uPA-eEVs) to enhance the tumor-targeting affinities. Consequently, in vivo, the systemic administration of uPA-eEV-PNCs nanococktail offered remarkable tumor regression and elongation of progression-free survival, providing evidence for the combinational antitumor effects of ncRNAs and engineered exosomes.^175^

CRISPR/Cas9 is confirmed to efficiently target specific parts of the genome, achieving encouraging outcomes. Although CRISPR/Cas9 is a potent therapeutic tool, delivering this editing tool in vivo specifically and safely is still challenging. Besides, it is still impossible to make sure CRISPR/Cas9 attacks all cells within the targeted pool. However, exosomes-loaded CRISPR/Cas9 was reported to address the above problems and activate necroptosis within tumor specifically.^176^

In a word, the therapeutic effects of ncRNA and CRISPR/Cas9 have been proven in spite of some issues. Engineered exosomes have shortened the gap between preclinical and clinical trials of ncRNA and CRISPR/Cas9-mediated gene therapy due to their high tumor-targeting ability and biocompatibility.

### Immunotherapy

Engineered exosomes derived from immune cells and tumor cells, serving as immune mediators, have attracted increasing attention owing to the inherent characteristic of exosomes (Table 2).

### Immunological engineered exosomes

Monoclonal antibodies (mAb) targeting specific molecules could be expressed on the surface of exosomes, which are potent “weapon” to activate antitumor immune response. For instance, the exosomes were modified with two different types of surface-displayed mAb, anti-human CD3 and anti-human HER2 antibodies, resulting in remarkably efficient and precise antitumor immune response both in vitro and in vivo.^56^ Apart from CD3 and HER2, PD1 and PDL1 are the classical immunosuppressive molecules. The anti-PD1 or anti-PDL1 antibodies have achieved unprecedented success in the field of antitumor immunotherapy. However, systemic administration of anti-PD1 or anti-PDL1 antibodies may lead to various immune-related adverse events (irAEs), including autoimmune diseases.^177^ The engineered exosomes loaded with therapeutic PD1- or PDL1-associated molecules showed similar tumoricidal effects on cancer cells with no severe irAEs.^124,125,178,179^ Recently, a new kind of engineered exosomes, the chimeric antigen receptor T (CAR-T) cell-inspiring exosomes, have attained a lot of attention due to the more antitumor effects and fewer irAEs, such as CAR-T-related encephalopathy syndrome (CRES)^180^ and cytokine release syndrome (CRS),^181^ compared with common exosomes and CAR-T therapy.^55,182^ In addition to mAb and CAR-T-cell-inspiring exosomes, immune adjuvant-loaded exosomes also trigger strong immune responses.^67,93,183^ Therefore, immunological engineered exosomes not only activate immune activity, but also avoid the uncontrollable irAEs, which is beneficial for clinical translation.

### Tumor-derived engineered exosomes serving as tumor vaccine

Tumor-derived natural exosomes inherit a number of molecules and factors from tumor cells, including MHC class I and II molecules, co-stimulatory molecules, and various cancer-specific antigens.^184^ Tumor-derived exosomes were proven to present tumor antigens to cytotoxic T cells, trigger the antitumor effects of T cells, and inhibit tumor growth in vivo.^185^ However, tumor-derived natural exosomes may lead to immune cell suppression and the escapes of immune surveillance, which extremely constrain the clinical application of natural exosomes.^186^

Given the above limitation, engineering modification provides a glimpse of hope for the utilization of tumor exosomes. Tumor-derived exosomes after proper modification, serving as antitumor vaccine, not only inherit molecules and tumor antigens, but also lead to more powerful antitumor immune activity and lower protumorigenic effects. For example, Ce6-loaded pancreatic cancer-derived re-assembled exosomes (Ce6-R-Exo) increased the release of cytokines from immune cells and consequently enhance the efficacy of immunotherapy under the guide of photoacoustic imaging.^187^ Ce6-R-Exo demonstrated that specific tissue-selective treatment via a laser is possible, which greatly minimized side effects in distant and normal tissues. Furthermore, long-term immunotherapy might be achieved by the presentation of antitumor vaccine as antigens to immune cells. The combination of photodynamic and immune therapy may be effective not only for primary treatment of solid tumors, but also for preventing recurrence and tumor metastasis. Furthermore, the nuclei from tumor cells were introduced into activated M1-like macrophages to generate chimeric exosomes (aMT-exos).^92^ These exosomes, combined with anti-PD1 treatment, prime T-cell activation via both the classical antigen-presenting cell–induced immunostimulatory approach and a unique “direct exosomes interaction” approach. Additionally, the immunosuppression in TME is remarkably alleviated after homing to tumor sites.^92^

In conclusion, the inherent tumor antigens endow tumor exosomes with advantages for antitumor vaccine. Nevertheless, tumor-derived exosomes, serving as therapeutic agents, are double-edged swords. There still exist multiple problems needed to be resolved, such as the immune cell suppression,^188^ the escapes of immune surveillance^189^ and the formation of pre-metastatic niche.^190–192^ Therefore, sorting and characterizing the specific subset of tumor-derived exosomes with high immunogenicity and low protumorigenic property is essential for the clinical translation of tumor-derived engineered exosomes.

### Engineered exosomes remodeling TME

**Fig. 5:**
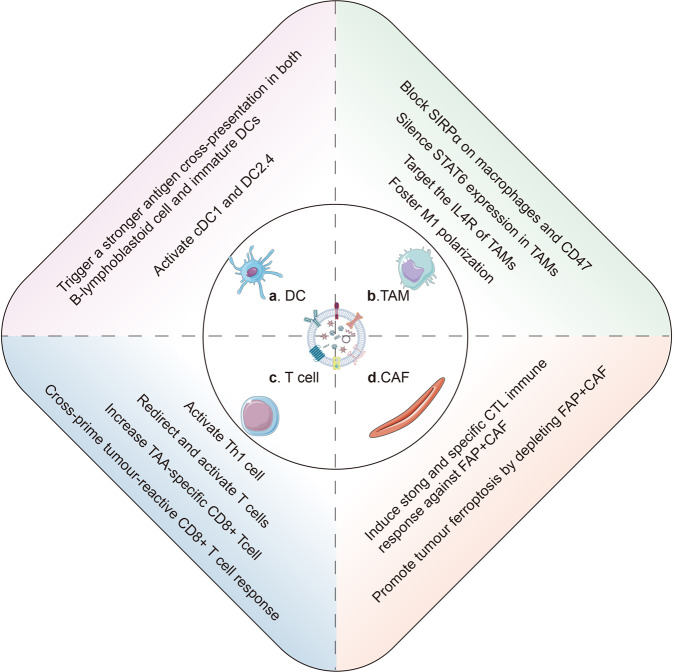
The engineered exosomes remodel the suppressive tumor microenvironment. **a** The internalization of engineered exosomes not only activates cDC1 and DC2.4, but also trigger a stronger antigen cross-presentation in both B-lymphoblastoid cells and monocyte-derived immature DCs. **b** Generally, the engineered exosomes reprogram tumor-associated macrophages from M2 to M1. Besides, the SIRPα and CD47 on macrophage could be blocked by the engineered exosomes. Specifically, exoASO-STAT6 could selectively silence STAT6 expression in TAMs. **c** In TNBC, engineered exosomes could prime and trigger T cells toward killing EGFR-positive TNBC. Additionally, the engineered exosomes could expand and cross-prime TAA-specific CD8 + T cells and activate Th1 cells. **d** Engineered exosomes induce strong and specific CTL immune response against tumor cells and FAP + CAFs. Furthermore, by releasing IFN-γ and depleting FAP + CAFs, the engineered exosomes could promote tumor ferroptosis

Mounting studies have proven that tumor immunosuppressive microenvironment significantly undermined the effectiveness of immunotherapy. Therefore, exploiting engineered exosomes to remodel tumor immunosuppressive microenvironment has been paid increasing attention to (Fig. 5).

Tumor-associated macrophages (TAMs) with an M2 phenotype are the major subgroup of suppressive immune cells. Reprogramming macrophages from an M2-like phenotype to an M1-like phenotype is proposed to be a realistic strategy to reverse tumor immunosuppressive microenvironment by engineered exosomes alone^88,193,194^ or with other therapy.^90,195^ For example, signal transducer and activator of transcription 6 (STAT6) is a key transcription factor to control M2 phenotype.^196^ M1 macrophage-derived exosomes were loaded with STAT6-targeting antisense oligo nucleotide (ASO), termed as exoASO-STAT6. In syngeneic models of CRC and HCC, exoASO-STAT6 alone induces nitric oxide synthase 2 (NOS2) expression, an M1 macrophage marker, leading to remodeling of the tumor immunosuppressive microenvironment and activating of a CD8 T-cell–mediated adaptive immune response.^88^

In addition to M2-like TAMs, fibroblast activation protein-α (FAP)^+^ cancer-associated fibroblasts (CAFs) serve as the target of engineered exosomes to remodel TME. For example, the FAP gene–targeting tumor-derived exosomes-like nanovesicles (eNVs-FAP) could trigger potent and specific cytotoxic T lymphocyte (CTL) immune responses against tumor cells and FAP^+^ CAFs and remodel immunosuppressive TME in multiple models. Furthermore, eNVs-FAP–activated immune responses could initiate tumor ferroptosis by releasing interferon-gamma (IFN-γ) from CTLs and depleting FAP^+^ CAFs.^51^

Collectively, in past decades, much progress has been made in understanding the contents and mechanisms of tumor immune microenvironment, which provides an enormous number of potential targets for engineered exosomes. Screening and validating the most suitable targeting immune cells or signaling molecules by integrating single-cell multiomics analyses^197^ remains a critical unresolved issue in the field of engineered exosome-based immunotherapy.

## Conclusion

Accumulated evidence has shown that exosomes could serve as a drug delivery system, which already has been evaluated in plenty of preclinical studies and attained encouraging results. However, natural exosomes also have several disadvantages, whose heterogeneity not only attenuates therapeutic efficacy, but also leads to tumor progression.

In order to address these problems, the engineering modifications of exosomes become essential. Unlike natural exosomes, engineered exosomes with membrane modification possess specific tumor-homing properties. Additionally, combining exosomes with other nanomaterials, such as liposomes^198^ and PNCs,^175^ also achieves enhanced or synergistic therapeutic effects. Most importantly, the development of bioinspired or biomimetic exosomes has shed light on the clinic-staged exosome-based drug delivery platform.^199^ Although compared with natural exosomes, engineered exosomes as drug delivery platform have a lot of advantages, there are also some challenges in the clinical translation of engineered exosomes. First, there still lack consensus on the standardization of methods for the isolation, quantification, and analysis of clinic-staged engineered exosomes from complex tissues, such as blood, tissue, and urine. Secondly, how exosomes and their contents should be exactly quantified, with options including exosome number, protein content, a ratio of the two,^200^ or classical mAb microarray-based surface profiling,^20^ remains elusive. Finally, since different sources of exosomes possess different functions and constituents, how to choose the suitable and available exosomes? These above problems should be resolved before developing exosome-based drug delivery system in clinical settings. Looking forward, because of such mounting evidences of the translation potential of engineered exosomes, immune cell-derived, nanomaterial-encapsulated, and aptamer-coated exosomes are hopefully to couple with external US irradiation for cancer-targeted therapy in the future.
